# Syphilis oculaire associée au VIH : À propos de 2 cas traités à Marrakech, Maroc

**DOI:** 10.48327/mtsi.v2i2.2021.181

**Published:** 2022-06-27

**Authors:** Hajar EL FOUAR, Khadija DANAOUI, Fatima IHBIBANE, Noura TASSI

**Affiliations:** Service des maladies infectieuses, Centre hospitalier universitaire Mohammed VI, Avenue Ibn Sina Amerchich, BP 2360 Principal, 40080 Marrakech, Maroc

**Keywords:** Uvéite, Papillite, VIH, Neurosyphilis, Pénicilline G, Ceftriaxone, Hôpital, Maroc, Maghreb, Afrique du Nord, Uveitis, Papillitis, HIV, Neurosyphilis, Penicillin G, Ceftriaxone, Hospital, Morocco, Maghreb, Northern Africa

## Abstract

**Introduction:**

Une recrudescence des cas de syphilis est observée depuis plusieurs années dans de nombreux pays, surtout en ce qui concerne les sujets infectés par le VIH. Ces patients se présentent souvent avec des lésions simultanées de syphilis primaire et secondaire, ou bien avec des formes étendues de syphilis.

**Observations:**

Nous rapportons 2 observations d'uvéite syphilitique prises en charge au service de Maladies infectieuses du Centre hospitalier universitaire Mohammed VI de Marrakech. La syphilis oculaire était inaugurale chez les 2 patients séropositifs. Malheureusement, aucun d'eux n'a bénéficié d'une récupération ophtalmologique complète.

**Conclusion:**

Devant toute uvéite ou papillite, il faut évoquer une infection syphilitique, d'autant que l'incidence de cette maladie est en augmentation depuis une dizaine d'années. Le traitement de la syphilis oculaire est mal codifié mais permet parfois une récupération fonctionnelle complète, s'il est précoce et adapté. La ceftriaxone pourrait être une alternative thérapeutique efficace à la pénicilline G dans le traitement de la syphilis précoce chez les individus infectés par le VIH.

## Abstract (en)

**Introduction:**

Syphilis is a sexually transmitted disease. All organs might be affected, but ocular syphilis occurs only in 0.6 percent of patients. A resurgence of syphilis cases has been observed for several years in many countries, especially in HIV-infected subjects. These patients often present with concomitant primary and secondary lesions or extensive presentations of syphilis.

**Case reports:**

We report 2 patients with syphilitic uveitis diagnosed and treated at the department of infectious diseases at the University hospital of Marrakech. Ocular involvement was inaugural in both HIV patients. Each had a specific treatment, but none had a complete recovery of visual function; the first patient was treated by ceftriaxone and the second one was treated by penicillin.

**Conclusion:**

Syphilis must be discussed in all patients diagnosed with uveitis or papillitis. The diagnosis should be suspected in cases of eye inflammation even in the absence of favourable clinical presentation or anamnesis. Search for HIV co-infection should be systematic. Although not evidence-based, prompt therapy may lead to functional recovery. Ceftriaxone could be a suitable alternative to penicillin in the treatment of early syphilis in HIV-infected patients. This treatment has a concomitant effectiveness even for asymptomatic forms of neurosyphilis. Ocular syphilis is a form of neurosyphilis and requires neurosyphilis therapy regardless of when it develops after primary infection.

Conventional syphilis staging is of little use in understanding ocular syphilis. Co-infection between HIV and ocular syphilis is common, but does not affect response to a neurosyphilis regimen of penicillin in the short term.

## Introduction

La syphilis, infection liée à *Treponema pallidum,* est en recrudescence depuis plusieurs années dans de nombreux pays. En France par exemple, elle touche en grande majorité les hommes, particulièrement chez les sujets infectés par le VIH [7, 9]. Selon l'Institut de veille sanitaire français (INVS), ils représentent 84 % des cas de syphilis diagnostiqués en 2014 en France et leur nombre a augmenté de 50 % entre 2012 et 2014 [9]. Au Maroc, en 2016, une modélisation avait estimé la prévalence de la syphilis à 0,4 % de la population adulte de 15 à 49 ans [10]. L'incidence de la neurosyphilis est élevée : 31 cas par an en 1985; elle a diminué à partir de 1990 pour atteindre 10 cas en 1997 [18].

Une classification plus thérapeutique distingue 2 groupes [5]. D'une part la syphilis précoce ou récente de moins d'un an d’évolution (syphilis primaire, secondaire, latente de moins d'un an) présentant une forte contagiosité et un faible risque de séquelles neurologiques, ne nécessitant qu'une injection d'extencilline. D'autre part la syphilis tardive (syphilis latente de plus d’1 an et syphilis tertiaire) avec faible contagiosité et fort risque de séquelles neurologiques [5], nécessitant 3 injections (en dehors de la neurosyphilis qui nécessite un traitement intraveineux pendant 14 jours). La neurosyphilis n'est pas forcément tertiaire et comprend notamment la syphilis oculaire. Celle-ci survient principalement au cours du stade précoce (secondaire ou latente) [2]. « Grande simulatrice », elle peut prendre toutes les formes d'uvéite. L'uvéite antérieure est présente chez 4 % des patients au stade secondaire et est bilatérale dans 50 % des cas [2]. Cependant, l'atteinte oculaire la plus fréquente est l'uvéite postérieure [1]. Les uvéites syphilitiques sont plus fréquentes chez les patients co-infectés par le VIH [4].

Nous rapportons ici 2 cas de syphilis avec atteinte oculaire, pris en charge dans le service de maladies infectieuses du Centre hospitalier universitaire Mohammed VI de Marrakech.

## Observation 1

Un jeune adulte âgé de 25 ans, homosexuel, sans antécédents pathologiques notables, consulte en ophtalmologie pour des troubles visuels profonds bilatéraux.

**Figure 1:**
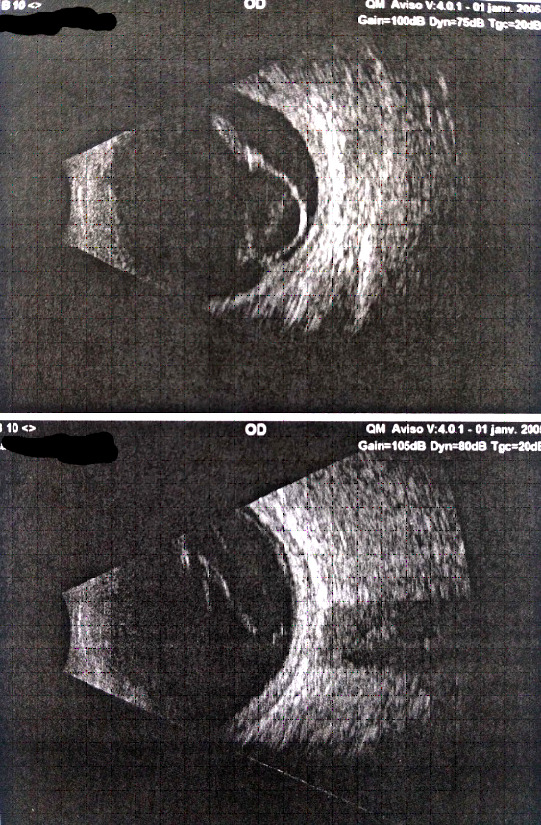
Échographie mode B objectivant une hyalite droite avec décollement de rétine en V. Collection Dr El Fouar, CHU Mohammed VI, Marrakech, Maroc. B-Mode ultrasound objectifying a right hyalitis with V-retinal detachment. Collection Dr El Fouar, CHU Mohammed VI, Marrakech, Morocco.

**Figure 2:**
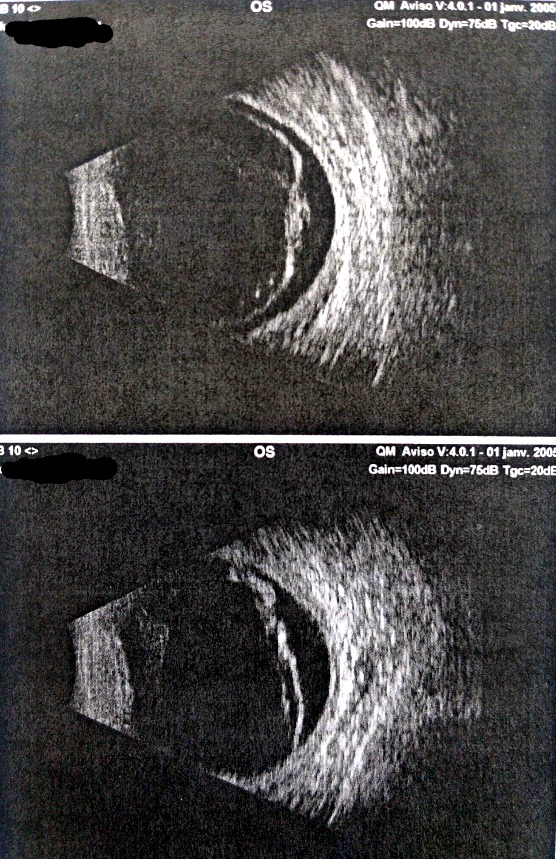
Échographie mode B objectivant une hyalite gauche avec décollement de rétine total gauche. Collection Dr El Fouar, CHU Mohammed VI, Marrakech, Maroc. B-Mode ultrasound objectifying a left hyalitis with total retinal detachment. Collection Dr El Fouar, CHU Mohammed VI, Marrakech, Morocco.

Le début de la symptomatologie semble remonter à 1 an et 3 mois par l'apparition d'un chancre induré, indolore, propre et bien limité au niveau du gland, évoquant une syphilis primaire, qui a spontanément guéri après 15 jours. Huit mois après, l’évolution a été marquée par la survenue d'une éruption maculo-papuleuse roséoliforme sur le dos et au niveau de la paume des deux mains évoquant une syphilis secondaire. Cinq mois après, le patient a présenté une douleur oculaire droite associée à une baisse de l'acuité visuelle (AV) homolatérale d'installation rapidement progressive (AV initiale de l’œil gauche était de 8/10^e^ et 5/10^e^ pour l’œil droit). Une semaine après, la symptomatologie s'est aggravée par l'installation d'une baisse de l'acuité visuelle gauche et une cécité à droite (AV de l’œil gauche était de 3/10^e^ et 0/10^e^ pour l’œil droit). Devant ce tableau, un fond d’œil a été réalisé objectivant une hyalite bilatérale. Une échographie oculaire en mode B a également été réalisée, objectivant une hyalite droite avec décollement de rétine en V droit et une hyalite gauche avec décollement de rétine total gauche (Fig. 1 et Fig. 2).

Le bilan biologique montre des leucocytes polynucléaires et lymphocytes sanguins à des taux normaux, une protéine C réactive (CRP) à 6,7 mg/ml, un *Treponema pallidum* hemagglutination assay (TPHA) positif à 10 240 et un Venereal disease research laboratory (VDRL) positif à 256. La sérologie du VIH est positive confirmée par le Western blot et un taux de CD4 à 403/mm^3^. La sérologie du VHB et VHC est négative. La sérologie CMV, IgG positive à 128 et IgM négative, et celle de la toxoplasmose est négative.

Un bilan de tuberculose a été demandé : GeneXpert dans les expectorations était négatif de même que la recherche de *Mycobacterium tuberculosis* à l'examen direct et la culture était négative.

L'examen du liquide céphalorachidien montrait une méningite lymphocytaire (60 % des lymphocytes) aseptique, une hyperprotéinorachie à 0,6 g/l et une hypoglycorachie (0,42/1,09 : 38,5 % de la glycémie). La PCR par la technique de FilmArray était négative. La recherche de bacille de Koch et le GeneXpert dans le LCR (liquide céphalo-rachidien) étaient négatifs. La sérologie syphilis dans le LCR montrait un TPHA positif à 1/1 280 et un VDRL négatif.

Le patient a reçu un traitement par céphalosporine orale de 3^e^ génération (C3G) 2 g/j en IV (intraveineuse) pendant 14 jours en raison de l'indisponibilité de pénicilline G, associé à un bolus de Solumédrol^®^ 500 mg/j en IV pendant 3 jours puis passage à la corticothérapie par voie orale à la dose de 1 mg/kg/j pendant 14 jours. Par la suite, le patient a été traité comme syphilis latente tardive par la pénicilline G à dose de 2,4 MUI (millions d'unités) par semaine pendant 3 semaines avec une amélioration de l'acuité visuelle au niveau de l’œil gauche coté à 8/10^e^, moins bonne à droite avec 3/10^e^, et une régression totale de l'hyalite et du décollement rétinien. Sur le plan biologique on note la négativation du VDRL dans le sang après 6 mois de traitement.

## Observation 2

Un jeune adulte âgé de 23 ans, hétérosexuel à partenaires multiples, suivi dans notre formation pour infection à VIH confirmée par Western blot révélée par une hyalite bilatérale. Avec un taux de CD4 initial à 327/mm^3^, il avait été mis sous traitement anti-rétroviral à base d'Atripla^®^.

À l'interrogatoire, le patient se plaignait de baisse de l'acuité visuelle avec yeux rouges douloureux bilatéraux. Le fond d’œil objectivait une hyalite bilatérale. Hospitalisé en médecine interne, le patient a reçu des bolus de corticoïdes sans amélioration. Il rapportait également la notion de chancre syphilitique non traité 1 an auparavant.

À l'examen clinique, on notait la présence des lésions palmo-plantaires à type de syphilide. Le reste de l'examen clinique était sans particularité.

Le bilan initial est revenu positif pour la syphilis : TPHA positif à 1280 et un VDRL faiblement positif.

L'examen ophtalmologique a montré une panuvéite bilatérale granulomateuse. Une échographie oculaire en mode B a objectivé un décollement rétinien postérieur incomplet en V des deux yeux.

Le bilan biologique complet a retrouvé des leucocytes, polynucléaires et lymphocytes sanguins à des taux normaux, une CRP à 0,52 mg/ml. La sérologie du VHB et VHC était négative. La sérologie CMV, IgG positive à 250 et IgM négative, et celle de la toxoplasmose était négative.

La recherche du GeneXpert dans les expectorations était négative comme celle de Mycobacterium tuberculosis à l'examen direct et en culture.

L'examen du liquide céphalorachidien était normal : aspect clair, 4 éléments leucocytaires, protéinorachie à 0,23 g/l et une glycorachie à 0,59 (glycémie concomitante à 1,09, rapport à 1,54). La sérologie syphilis dans le LCR montrait un TPHA faiblement positif et un VDRL négatif.

Le patient a reçu un traitement par pénicilline G : 25 MUI par jour en 6 perfusions pendant 14 jours associé à un bolus de Solumédrol pendant 3 jours, puis passage à la corticothérapie par voie orale à la dose de 1 mg/kg/j pendant 14 jours. Par la suite, le patient a été traité comme syphilis latente tardive par la pénicilline G à dose de 2,4 MUI par semaine pendant 3 semaines. L’évolution était caractérisée par une amélioration rapide des lésions cutanées et oculaires, avec en quelques jours la quasi-disparition des syphilides et une très nette diminution de l'inflammation de la chambre postérieure au fond d’œil et un début de récupération d'acuité visuelle (AV de l’œil gauche à 6/10^e^ et 7/10^e^ pour l’œil droit). Sur le plan biologique, le VDRL était négatif dans le sang après 6 mois de traitement.

## Discussion

Les atteintes oculaires de nos 2 patients étaient différentes dans leur présentation et leur gravité. La sérologie syphilitique doit être réalisée systématiquement devant une uvéite ou bien une hyalite d’étiologie incertaine, mais certains malades peuvent y échapper, notamment en l'absence de manifestations extra-oculaires évocatrices et même d'un terrain à risque d'IST (infection sexuellement transmissible) [8].

La prise en charge de l'atteinte oculaire de la syphilis n'est pas parfaitement codifiée. Selon les recommandations de 2010 sur la prise en charge des maladies sexuellement transmissibles (Sexually transmitted diseases treatment guidelines, MMWR 2010) [17], les atteintes oculaires de la syphilis doivent être traitées selon les protocoles appliqués dans la neurosyphilis, car la rétine et le nerf optique sont considérés comme étant un prolongement du système nerveux central [8]. Cependant, ces recommandations, fondées sur plus de 50 ans d'expérience médicale, ne reposent pas sur des essais cliniques standardisés.

Dans la littérature, le traitement de la névrite optique et de la rétinite est assez consensuel mais celui des uvéites fait encore débat [8]. Les protocoles européens recommandent la benzathine pénicilline en intraveineuse, à une dose totale de 18 à 24 MUI (millions d'unités) pendant 10 à 14 jours [8]. Cependant, dans l’étude d'Amaratunge *et al.* [1, 6], parmi les 13 cas d’échec du traitement, 11 avaient reçu de la pénicilline en intraveineuse. Certains auteurs utilisent d'autres stratégies thérapeutiques avec de bons résultats. Tieulié *et al.* [14] ont traité deux cas d’œdème papillaire par de la ceftriaxone, avec une évolution favorable comme le cas de notre seconde observation. Puech *et al.* [13] ont montré une bonne efficacité de la ceftriaxone à la dose de 1 g par jour pendant 3 semaines chez 7 patients avec différentes atteintes oculaires. Selon Psomas *et al.* [12], la ceftriaxone et la doxycycline pourraient être des alternatives thérapeutiques efficaces à la pénicilline dans le traitement de la syphilis précoce chez les individus infectés par le VIH-1, comme pour notre premier patient. Le bénéfice supplémentaire de ces régimes thérapeutiques pourrait consister, contrairement à la pénicilline, dans leur activité simultanée sur les formes asymptomatiques de neurosyphilis.

L'utilisation de corticoïdes en association à la pénicilline dans les syphilis oculaires n'est pas mentionnée par les dernières recommandations. Pourtant, selon l’étude de Prokosch et Thanos de 2008 [11], ce traitement pourrait être prometteur en termes de récupération dans les cas de névrite optique. Enfin, afin de prévenir la réaction de Jarisch-Herxheimer, possible en début de traitement notamment dans la syphilis primaire, les recommandations de 2010 (Sexually transmitted diseases treatment guidelines, MMWR 2010) [17] proposent d'utiliser des antipyrétiques, sans qu'il y ait eu de preuve de leur efficacité [8]. Danesh-Meyer *et al.* proposent l'utilisation de corticoïdes [3].

Pour ce qui concerne le suivi des syphilis oculaires, en plus de la surveillance sérologique à6et12 mois comme dans les stades primaire et secondaire, il est recommandé de réaliser une nouvelle ponction lombaire tous les 6 mois si la précédente était anormale [4]. Cependant, peu de données démontrent que la normalisation de la sérologie va de pair avec la normalisation du LCR. La protéinorachie et le VDRL dans le LCR mettent plus de temps à se normaliser. Il convient de recommencer un traitement si le taux de cellules n'a pas diminué à 6 mois ou si la protéinorachie ne s'est pas normalisée à 2 ans.

L'augmentation de l'incidence de la syphilis impose de la rechercher systématiquement devant toute uvéite, même antérieure non granulomateuse d'apparence bénigne, afin d’éviter une administration intempestive de corticoïdes sans couverture antibiotique. Sa détection précoce permet aussi une récupération visuelle optimale [8]. Après un traitement bien codifié, seulement 10 % des cas ont présenté des séquelles visuelles [2]. L'atteinte ophtalmologique peut être l'atteinte initiale isolée et peut récidiver jusque dans 14 % des cas malgré un traitement optimal et précoce, la plupart du temps par persistance des facteurs de risque d'IST [15, 16].

Un diagnostic erroné devant les manifestations oculaires de la syphilis ou un diagnostic et un traitement retardés peuvent conduire à une perte de vision irréversible.

la ceftriaxone est aussi efficace. Cependant, des études sur de plus grandes populations seraient nécessaires afin d'aboutir à des recommandations thérapeutiques plus précises et plus spécifiques des différentes atteintes oculaires qui peuvent être observées au cours de la syphilis. L'intérêt éventuel d'une corticothérapie doit également être étudié. Le diagnostic rapide de cette cause rare d'uvéite est d'autant plus important qu'un traitement simple et efficace est disponible.

## Conclusion

Dans le contexte d'une augmentation récente de l'incidence de la syphilis, il faut évoquer cette maladie devant toute atteinte inflammatoire oculaire, y compris en l'absence de signes cliniques extra-oculaires. Même si l'infection syphilitique peut survenir chez le patient immunocompétent, une co-infection par le VIH est fréquente et doit être recherchée. Le traitement de l'uvéite syphilitique est relativement bien codifié et repose principalement sur la pénicilline G en intraveineuse, mais une alternative par

## Liens D'intérêts

Les auteurs ne déclarent aucun lien d'intérêt.

## Contribution Des Auteurs

H. El Fouar : Rédaction du manuscrit et réalisation des illustrations

K. Danaoui : Recherche bibliographique

F. Ihbibane : Relecture et correction du manuscrit

N. Tassi : Coordination de la rédaction du manuscrit, relecture et correction du manuscrit.
